# Informatics Approach Towards Targeting HTR1B Pathways in Neuropharmacology for Migraine Treatment

**DOI:** 10.2174/011570159X341703250130064735

**Published:** 2025-02-06

**Authors:** Saleem Ahmad, Li Wang, Imran Zafar, Zain Abbas, Ahsanullah Unar, Mohamed Mohany, Salim S. Al-Rejaie, Najeeb Ullah Khan, Ijaz Ali, Muhammad Shafiq

**Affiliations:** 1 Cardiovascular Center of Excellence, Louisiana State University Health Sciences Center, New Orleans, LA, United States;; 2 Shenzhen Hospital Beijing University of Chinese Medicine, Guangdong, China;; 3 Shenzhen University General Hospital, Shenzhen University, Guangdong, China;; 4 Department of Biotechnology, The University of Faisalabad (TUF), Faisalabad, Punjab, Pakistan;; 5 Department of Life Sciences, University of Management and Technology, Lahore, Punjab, Pakistan;; 6 Department of Precision Medicine, University of Campania ‘L. Vanvitelli’, Naples, Italy;; 7 Department of Pharmacology and Toxicology, College of Pharmacy, King Saud University, P.O. Box 55760, Riyadh 11451, Saudi Arabia;; 8 Institute of Biotechnology & Genetic Engineering (Health Division), The University of Agriculture, Peshawar, Pakistan;; 9 Centre for Applied Mathematics and Bioinformatics (CAMB), Gulf University for Science and Technology, Hawally, Kuwait;; 10 Research Institute of Clinical Pharmacy, Department of Pharmacology, Shantou University Medical College, Shantou, 515041, China

**Keywords:** HTR1B pathway, molecular docking, network pharmacology, pharmacokinetic profiling, molecular dynamics simulation, migraine treatment

## Abstract

**Introduction:**

Migraine is a prevalent and debilitating neurological disorder, with current therapies are frequently ineffective and have side effects. Recent studies in neuropharmacology present the serotonin 1B receptor (HTR1B) receptor as a viable avenue of migraine treatment since it influences pain and vasoconstriction.

**Methods:**

This research broadly uses computational approaches to explain the 5-hydroxytryptamine receptor 1B (HTR1B) pathways in neuropharmacology for migraine treatment.

**Results:**

Text mining results reveal 25 essential genes, and network pharmacology provides complex mechanisms among genes and proteins, revealing a sophisticated network consisting of 41 nodes and 361 edges. The protein structure and function were elucidated through high-resolution protein modelling and validation, yielding significant new information. The structure has a resolution of 2.05 Å and a C-score of 0.30. The virtual screening explored the best ligands, which had binding affinities ranging from -13.8 to -9.6 kcal/mol from a set of 25 molecules. Docking results indicated that FDA-approved ligands showed high binding affinities, ranging from -11.4 to -12.5 kcal/mol among other natural and synthetic libraries. The pharmacokinetic profiles of the potential drugs showed significant diversity in their solubility and lipophilicity qualities (F(2,6) = 15.13, *p* = 0.004), suggesting different levels of safety and efficacy. MD simulation clarified the dynamic interactions between the protein and ligand at 100ns. The RMSD values were stable within the 6.0-7.5 Å range, indicating a consistent structure. RMSF values revealed areas of flexibility in the protein. The toxicity risk assessment of Xaliproden indicated modest risks.

**Conclusion:**

This study provides a foundation for targeted HTR1B-based migraine therapies and highlights the value of informatics tools in accelerating drug discovery in neuropharmacology.

## 1 INTRODUCTION

The migraine is a chronic neurological disorder that manifests through severe headaches and affects a large portion of the population of the world; it is the second leading cause of years lived with disability (YLD) [1, 2]. Posted treatments are frequently insufficient, and a new therapeutic approach is necessary [1, 2]. Migraine pathophysiology is associated with the 5-hydroxytryptamine receptor 1B (HTR1B), a serotonin receptor subtype. By modulating serotonin levels and migraine perceptions and triggers, it controls migraine intensity and tenderness. HTR1B also controls pain-transmitting pathways by preventing neurotransmitter release, thus causing vasoconstriction [3-5]. Situated in the brain areas involved in pain modulation, both HTR1B agonist effects might help in lowering pain sensations and modulating pro-inflammatory signaling in migraines [6, 7]. It is pivotal to comprehend the function of HTR1B to discover efficient, individualized medicinal approaches to migraine [6].

Several migraine susceptibility and treatment response genes, including SLC6A4, code for serotonin transporter and influence serotonin reuptake, and COMT, which encodes for catechol-O-methyltransferase, affecting the degradation of catecholamines interact with the serotonin system and the HTR1B receptor [8]. Polymorphisms in genes that encode serotonin metabolism are MAOA, TPH1, and TPH2; these may also influence migraine and its management [9]. Further, alteration in TRPM8 may affect the pain pathways for migraine and mutations in CACNA1A linked with FHM. Although these genetic factors are not associated with HTR1B [10], understanding serotonin signaling and migraine helps develop pharmacotherapeutic approaches [11]. The preferably selective 5-HT1B agonist is Xaliproden, which can cause vasoconstriction in the cerebral vessels and have a stabilizing impact on the neurotransmitters, reducing the frequency and severity of migraines [12]. Sumatriptan and rizatriptan are triptans that work on 5-HT1B/1D receptors to alleviate acute migraine through vasoconstriction and inhibition of neurogenic inflammation [13], while ditans, including lasmiditan, act on 5-HT1F receptors and alternative that has fewer vascular side effects and is safer than triptans for patients who cannot tolerate.

The 5-HT1 receptor-mediated signal transduction pathway is critically involved in migraine therapy and risk [14, 15], as depicted in Fig. (**1**). Stimulation of 5-HT1 receptors leads to a decrease in the Intensity of pain signal and neuronal excitability following inhibition of adenylyl cyclase and cyclic adenosine monophosphate (cAMP) [14]. This receptor modulation also affects the efflux of neurotransmitters and neurotransmission [15]. The mitogenic stimulus of human astrocytes conveys increased 5-HT1 receptor activation, and balanced 5-HT1, and 5-HT2 receptors modulate neuronal function and migraine [16]. Thus, migraine pain pathway modulation could be related to ATP release and SNARE complex formation provoked by 5-HT1 receptor activation [17, 18]. The HTR1B pathway has advanced gradually, mainly owing to challenges posed by the multifaceted molecular nature of migraine and the shortcomings associated with the conventional drug development methodology [17, 18]. Traditional approaches are slow and can not exhaust the relationships between the factors implicated in the development of migraine. Computational methods provide a potential solution to the problem by allowing the high-throughput screening and ranking of novel drug candidates targeting the HTR1B pathway, accelerating the early stage of drug discovery and potential treatments’ designation [19-21].

In addition, traditional methods of recombinant-based drug searching are adopted for increased information on the complex structures and protein-protein interactions (PPINT) related to migraine formation [22]. These techniques eliminate the shortcomings of target-centric strategies dominating earlier and offer a global view of drug design. Using a network approach, they can define how the numerous molecules interact in complex ways to cause migraine [23]. Drug development based on the network concept offers enhanced and sustainable therapeutic solutions by posing greater importance to the bio-contextual understanding and complex interactions among many targets and pathways [24]. This approach is suitable for migraine mainly because this chronic headache disorder's development requires modulating several pathways and systems [25].

This research aims to enhance migraine treatments by exploring the interoperability of Network-Based Drug Repositioning and *in-silico* Screening. The comprehensive interactions by which these putative drugs operate are understood perfectly by employing molecular docking studies, computer simulations, and, most importantly, network analysis. Many of the identified potential candidates are then investigated for further experimental confirmation, and their pharmacological efficacy is assessed with the help of computational tools. These computational techniques shorten drug discovery time, enhance candidate identification, and thus enable promising therapy for migraines within the precision medicine framework. Combining computational approaches with traditional experimental practices may revolutionize the drug discovery process in neuropharmacology and boost the global potential of migraine patients.

## 2 MATERIALS AND METHODS

The study begins by exploring the function of HTR1B within the context of migraine, as depicted in Fig. (**2**). Critical insights are extracted and analyzed through advanced keyword mining and data retrieval, enabling a thorough evaluation of the receptor's role.

### 2.1 Data Collection and Text Mining

To investigate the involvement of the 5-hydroxytryptamine receptor 1B (HTR1B) pathway in the onset of migraine [17], we conducted a comprehensive literature review using PubMed (https://pubmed.ncbi.nlm.nih.gov/), Scopus (https://www.scopus.com/), and Google Scholar (https://scholar.google.com/*)* as our primary databases. We performed a systematic search using a string of relevant keywords to identify relevant articles, such as “HTR1B,” “migraine,” “neuropharmacology,” and “drug discovery” [7]. To ensure the validity and reliability of the data, we limited our selection to peer-reviewed articles available up to the time of the study [7]. The search results included a variety of sources such as reviews, case reports, and empirical studies, from which we selected 25 publications based on their quality and relevance and detailed information, as mentioned in Table **S1**.

The data extraction process was thus centered on identifying important information about the paths, interactions, and functions of HTR1B in migraine. Only publications reporting protein structure, function, or expression data that gave some information about the role of HTR1B in migraine formation were considered [26]. The selected texts were analyzed using systematic Natural Language Processing (NLP) approaches to obtain the data, as stated by [27]. Named Entity Recognition (NER) was utilized to extract key biological entities related to HTR1B, including genes, proteins, and chemical compounds described in the texts [28]. Tokenization and part-of-spoke (POS) tagging were then used to process the text further, segment it into words and phrases, and find the correct syntactic role of each word and relevant terms and their relations. Further, we applied the word embedding approach, including Word2Vec, to analyze the semantic associations of the terms in the systematic migration research. This enabled us to examine the relations and correlations the text could not see. To refine our understanding of the overarching themes, we employed Latent Dirichlet Allocation (LDA) for topic modeling, which helped identify and cluster the main themes across the corpus of selected articles [29]. These clusters were then subjected to hierarchical clustering of cluster-related topics, thereby gaining better insight into the focus areas within the literature. Since the natural language interpretation of text may contain unclear words and their meanings depend on the context, we used contextual disambiguation methods in our work. From examining the context of terms every time, we could exclude additional connotations, including those outside of neuropharmacology and migraine research, when defining essential terms. As per the informational retrieval protocol, network analysis from the STRING database (https://string-db.org/) revealed connections between HTR1B, various chemicals, and migraine-related physiological processes [6].

### 2.2 Network-based Drug Discovery Framework

We developed protein-protein interaction (PPI) networks for the pathophysiology of migraines using Cytoscape version 3.7.1 [30] and STRING (https://string-db.org/) [31] to generate and display the networks. We conducted a network-based analysis to determine which nodes and modules were most important for the pathway. We use clustering techniques using Cytoscape (3.7.1) [30] and NetworkAnalyzern (3.7.2) as an *in-silico* for centrality metrics and functional enrichment analysis to classify the most relevant nodes and pathways associated with migraine. Further, we validate our results using Excel and GraphPad Prism (10.3) to improve the accuracy of network-based predictions and discover new treatment targets.

### 2.3 Curation of 3D Structure of Therapeutic Target

We investigated the 3D structure of the HTR1B protein having the PDB ID: 2ANY, which is associated with migraine. For the conformation of different conformational changes in protein structure, we use PyMol Version (3.0) [32, 33] and align with a canonical sequence of the HTR1B protein (UniProtKB Entry No. P07288) to predict 3D structure. We submitted this sequence to the I-TASSER (Iterative Threading ASSEmbly Refinement) system [34], which produced ten different models. Each model was evaluated using the C-Score, which ranges from -5 to 2 and indicates the reliability of the predicted structures. The model with the highest C-Score of +1.75 was selected, reflecting high confidence in its accuracy [34]. For the verification of the validity of the chosen model, convergence parameters, such as RMSD, RMSF, energy minimization, stability, radius of gyration, distance metrics, and binding free energy were established to determine the stability of the structure at different points of the simulations, threading template alignment that contained structural information about homologous proteins was integrated.

### 2.4 Virtual Screening

We screen different libraries against the expected binding sites of HTR1B-bound proteins for computer-assisted drug development to target therapeutic candidates [35, 36] to find compounds with high binding affinities [37]. We use AutoDock Vina (version 4.2) as a screening tool to calculate binding scores [38]. Further, we optimize and prepare ligands using MTI Open Screen Suite (https://bio.tools/MTiOpenScreen) and AutoDock Vina [39] to analyze virtual screening results.

### 2.5 Molecular Docking Studies

We performed molecular docking simulations to predict binding interactions between certain drugs and HTR1B-related proteins [40, 41]. Three-dimensional structures were acquired from the Protein Data Bank (https://www.rcsb.org/) to construct protein structures, remove water molecules, insert missing atoms, and adjust protonation states [42]. AutoDock Vina (4.2) was used for molecular docking [43] to identify the binding site and grid box, generate ligand conformations, run docking simulations, and evaluate and classify docked poses. To assess the binding interactions among HTR1B-related proteins, including binding affinity (Kd), binding energy (ΔG), hydrogen bonding interactions, hydrophobic interactions, and electrostatic interactions, the results of molecular docking simulations were analyzed [44].

### 2.6 Profiling of Protein-ligand Interaction

We filtered the results to select candidates with binding affinities lower than -11 kcal/mol for further investigation. We used the Protein-Ligand Interaction Profiler (https://plip-tool.biotec.tu-dresden.de/plip-web/plip/index) to visualize and analyze non-covalent interactions between proteins and ligands, allowing us to comprehensively examine the interactions between the ligands and the therapeutic target [45, 46].

### 2.7 The Prediction of ADMETox and Bioavailability

We Predict ADMETox (absorption, distribution, metabolism, excretion, and toxicity) and bioavailability using the SwissADME platform (http://www.swissadme.ch/) for drug development. Drug similarity and pharmacokinetic properties were evaluated using Veber criteria [47] and Lipinski's rule of five [48]. The ADME/Tox profile was calculated using ADMETlab 2.0 [49] to predict pharmacokinetic and toxicological characteristics.

### 2.8 Molecular Dynamics (MD) Simulations

We conducted *in silico* molecular dynamics (MD) simulations to investigate the interaction between proteins and ligands [50]. The protein-ligand complex was generated using Schrödinger software, with the protein structure obtained from the Protein Data Bank (PDB). We used Glide's Extra Precision (XP) or Standard Precision (SP) docking methods, parameterized the ligand with LigPrep, and used the OPLS3 force field for system parameterization [51]. The system was solvated in a cubic box with water molecules with TIP3P (transferable intermolecular potential with 3 points) and balanced with counterions. MD simulations were performed under constant temperature (300 K) and pressure for 100 nanoseconds [52]. We assessed structural stability using RMSD (root mean square deviation) and RMSF (root mean square fluctuation) analyses of the docked conformers [53, 54].

### 2.9 Statistical Analysis

Statistical studies were performed using appropriate software packages, such as R (https://www.r-project.org/) and GraphPad Prism (https://www.graphpad.com/features), to evaluate experimental data and computational results. The experimental data were examined using graphical representations, significance testing, correlation analysis, and descriptive statistics to facilitate accurate conclusions and interpretation of the findings.

## 3 RESULTS

### 3.1 Text Mining Analysis

Our systematic literature review, which included 200 articles and focused on migraine comorbidities, identified the HTR1B pathway as an essential contender for developing migraine. We highlighted the critical role of HTR1B receptors in processes associated with neurotransmitter release, ion channels, and intracellular signaling regarding pain. Further, the analysis helped identify the treatment-related therapeutic spots HTR1B and genes linked to it, such as GNA1, MDFI, and GNO1. 25 genes control the pathway and these genes are CACNA1A, KCNK18, HTR1B1, EDNRA, FHL5, ATP1A2, HTR3D, HTR1B, KCNK15, RAMP1, ADM2, PRRT2, TGFB, SNAP25, ASTN2, MEF2D, GSTK1, MDFI, ACT. Fig. (**3**) also shows the extensive involvement of the HTR1B pathway in migraine processes.

### 3.2 Network Pharmacology

#### 3.2.1 Network Analysis of HTR1B Pathways in Migraine

We also observed that the G protein-coupled serotonin receptor 1B (HTR1B) is the most critical gene in migraine-related pathways due to participation in the complex signaling networks. HTR1B consists of membrane-bound receptors that change conformation with ligand binding to interact with G proteins and, subsequently, affect targets like adenylate cyclase. Arrestins can alter other signaling pathways by affecting the G protein-mediated signal. HTR1B recruits GNB1 and GNG2, which are inherent to G protein functionality, besides HTR1D, HTR1A, and HTR2A, which are conceived to share signal pathways. Neurotransmission on serotonergic pathways is determined by serotonin transporter SLC6A4, which modulates serotonin reuptake. The network analysis provided an interaction network with 361 edges and 41 nodes, an average local clustering coefficient of 0.716, and a node degree of 17.6 with a significant *p*-value <1.0E-16. This graph's expected number of edges is fifty, as shown in Fig. (**4**).

### 3.3 Protein Model Analysis and Validation

For protein model analysis and validation, we use in-silico X-ray diffraction to find the molecular structure with a resolution of 2.05 Å of HTR1B protein. The results demonstrate a high level of precision and reliability, with R values of work (0.192), free (0.236), and observed (0.194) to identify a solid foundation. The selected HTR1B model, which had a C-score of 0.30, was uploaded to the iMod server (https://imods.iqf.csic.es/) and displayed in four unique modes, as seen in Fig. (**5**). The selected model has high structural stability, as shown by a favorable C-score and average Z-score. Secondary structure representation often looks helical, highlighting possible binding locations and functional regions.

**Fig. (6):**
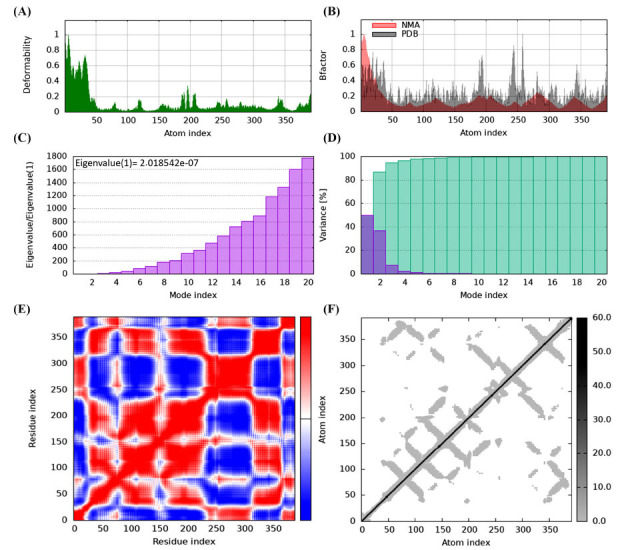
(**A**) Deformability vs. atomic index, highlighting protein flexibility, (**B**) B-factor *vs*. atomic index, showing thermal motion variations. (**C**) Eigenvalue analysis indicating structural stiffness. (**D**) Variance distribution reflecting structural adaptability. (**E**) Covariance map of residue correlations. (**F**) Elastic network model depicting stiffness and flexibility.

The selected model allows a comprehensive examination of the molecular structure. The deformability values of the residues tend to increase with higher atomic numbers, as shown by the positive correlation observed in the plot of deformability and atomic index (Fig. **6A**). The protein structure exhibits remarkable flexibility, as evidenced by the range of deformability values between 0.2 and 0.5. The self-analysis findings reveal that the protein structure exhibits significant flexibility, as demonstrated by the relatively high deformability scores. A closer analysis of the protein sequence reveals that areas exhibiting substantial sequence variability align with regions characterized by high deformability. This suggests that the protein could undergo conformational alterations to adapt to different binding partners. Residues with higher atomic numbers often exhibit higher B-factor values, as seen by the positive correlation between these two parameters in the plot of B-factor and atomic index (Fig. **6B**).

The B factor, or the Debye-Waller or temperature factor, is a critical parameter in crystallography that quantifies atoms' thermal motion or flexibility within a protein structure. The values of the B factor typically range from 0 to 1, where lower values indicate more rigid regions and higher values correspond to more flexible areas. The range of the MNA (Molecular Normal Mode Analysis) B factor, illustrated above, spans from 0.6 to 1, indicating a notable level of flexibility in this specific region. The PDB (Protein Data Bank) B factor ranges from 0.2 to 0.6, indicating a notable degree of flexibility in this region. The protein structure reveals that areas with high crystallographic temperature factors correspond to regions with high B factors. This suggests that the protein may undergo thermal motion to facilitate binding or catalysis. Fig. (**6C**) shows the eigenvalues of 2.01842e-07, representing the energy required to modify the level of stiffness and structure.

A lower eigenvalue means a lower degree of complex deformation. Deformation requires a specific amount of energy expenditure because the stiffness constant of the structure is 0.024. Fig. (**6D**) shows the variance associated with each normal mode, ranging from 0.01 to 0.1. Individual (red) and cumulative (green) fluctuations combine to form a total variance of 0.5, indicating the structure's adaptability and diversity level. Fig. (**6E**) presents a covariance map with a correlation coefficient of 0.7. The map represents the correlation between pairs of residuals, with values ranging between -0.5 and 0.5. The data displayed exhibits a positive correlation (red), no correlation (white), or a negative correlation (blue). Fig. (**6F**) shows the elastic network paradigm, which defines the relationships between atoms using springs. The model provides a deep understanding of structural dynamics, stiffness, flexibility, and relationships, with an average spring constant of 0.5. The stiffness of the points determines their tone. Points with higher grayscale values have stiffness values ranging from 0.1 to 1, while points with lighter values have stiffness values ranging from 0.01 to 0.1.

### 3.4 *In-Silico* Screening of Potential Drug Candidates

#### 3.4.1 Virtual Screening

A total of 2500 compounds were chosen from a chemical library for the *in silico* screening process. This collection had 600 FDA-approved pharmaceutical products, 1,200 natural products, and 700 synthetic substances. Compound selection was based on their chemical diversity, therapeutic development suitability, and structural information availability from the ChemSpider (https://www.chemspider.com/) and PubChem (https://pubchem.ncbi.nlm.nih.gov/) databases. The viral detection process identified very potent ligands. The binding affinities of the top 100 FDA-approved drugs with the target protein are shown in Table (**S2**). These affinities range from -13.8 to -11 kcal/mol. Vina-based virtual screening for food-derived small molecules yielded complexes with binding affinities ranging from -12.5 to -10.9 kcal/mol, as shown in Table (**S3**). Data presented in Table (**S4**) demonstrate that the top 100 ligands from the natural compound library had a binding affinity range of -13.2 to -9.6 kcal/mol with the target protein. To perform a deeper *in-silico* molecular docking study, we selected the top 10 interacting molecules from each data set, resulting in 30 molecules.

#### 3.4.2 Ligand-based Screen Interactions

Based on the ligand-based compound screening data in Table **1** and the 2D structure depicted in Fig. (**7**), aminohippuric acid emerges as the most promising candidate, with a maximum score of 0.553. This indicates a notable potential for biological activity or a strong attraction toward a specific chemical target. Aminobenzoic acid followed closely, achieving a score of 0.429, again indicating significant activity. Benzoic acid, with a score of 0.390, also shows considerable potential. Due to their substantially higher scores, suggesting favorable interactions with the target, these three lead compounds need further investigation. The ligand information provides crucial information about the chemical properties of these molecules. Aminobenzoic acid has two hydrogen bond donors, four hydrogen bond acceptors, and a molecular weight of 166.1. With a log *P* value of 0.9, it exhibits moderate lipophilicity and features a single ring containing four nitrogen and oxygen atoms, which potentially serve as binding sites. In contrast, aminohippuric acid has a molecular weight of 180.2 and a logP value slightly more significant than 2.3, indicating its greater hydrophobicity. Unlike aminobenzoic acid, this compound has two hydrogen bond acceptors and one hydrogen bond donor, suggesting unique characteristics in terms of binding.

For revealing ligands and receptors' strong affinities score, we use FitDock (http://cao.labshare.cn/fitdock/php/register.php), with amino hippuric acid standing out with a value of 0.87 shows high structural similarity to known ligands. Ligmate and Rigid-LS-align also demonstrate promising alignments but to varying degrees. Morgan Fingerprint, FP2, FP4, and Flexi-LS-align scores provide further information on potential pharmacophore matches and molecular similarities. Prime candidates are amino hippuric acid, aminobenzoic acid, and benzoic acid, which exhibit favorable screening scores and desirable ligand characteristics. Further computational modeling and experimental validations are recommended to understand these substances' pharmacological potential better and optimize their use in drug development projects.

#### 3.4.3 Structure-based Screen Analysis

**Fig. (8):**
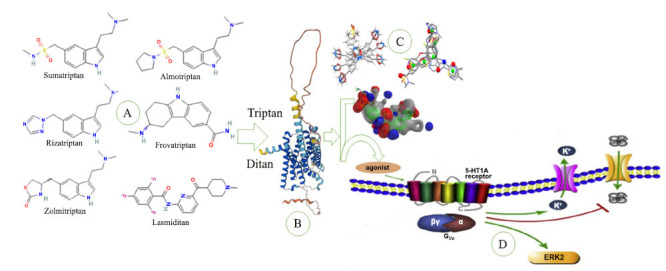
Triptans and ditans (**A**) interact with the HTR1B and 5-HT1F proteins (**B**), forming model complexes with vital binding energies and excellent RMSD values (**C**), specifically targeting migraine pathways by acting as agonists (**D**).

In our structure-based screening analysis, we evaluated the interactions of triptans and ditans with serotonin receptors critical for migraine treatment, as seen in Fig. (**8**). Triptans such as sumatriptan, rizatriptan, zolmitriptan, almotriptan, and frovatriptan target the 5-HT1B/1D receptors (Fig. **8A**), with rizatriptan showing the highest binding affinity (binding energy of -8.7 kcal/mol, RMSD of 1.1 Å), followed by zolmitriptan (-8.5 kcal/mol, RMSD 1.3 Å), almotriptan (-8.4 kcal/mol, RMSD 1.2 Å), sumatriptan (-8.3 kcal/mol, RMSD 1.2 Å), and frovatriptan (-8.0 kcal/mol, RMSD 1.4 Å) make complex model and show interaction in specific region as seen in Fig. (**8C**). Ditans like lasmiditan and Reyvow work on the 5-HT1F receptor associated with blocking the pain pathway and are free of vasoconstrictor effects, which can be dangerous for cardiovascular patients. Lasmiditan exhibited a binding energy of -9.2 kcal/mol with an RMSD of 1.0 Å. Reyvow demonstrated an even stronger binding affinity of -9.4 kcal/mol with an RMSD of 0.9 Å, as seen in Fig. (**8B**). These findings suggest that rizatriptan and ditans (lasmiditan and Reyvow), attached as agonists, may be particularly effective for acute migraine relief due to their strong receptor interaction, as shown in Fig. (**8D**).

### 3.5 Docking Results

Based on an analysis of these compounds (Table **2**), each FDA-approved ligand has a significant binding affinity, with values ranging from -11.4 kcal/mol (halopemide) to -12.5 kcal/mol(bolazine). The exact binding affinities of Xaliproden, Paliroden, Spirofilina, and Halopemide are -11.9 kcal/mol, -11.5 kcal/mol, and -11.4 kcal/mol, respectively. Furthermore, the compounds have significant lipophilicity, as demonstrated by their high LogP values, ranging from 1.5 for sporophylls to 9.2 for bolazine. Compounds vary in the number of hydrogen bond donors and acceptors. Xaliproden and Paliroden have no donors and four acceptors, Bolazine has two donors and four acceptors, Spirofilina has no donors and seven acceptors, and Halopemide has two donors and four acceptors. All compounds have high molecular weights, with Xaliproden weighing 381.4 g/mol and Bolazine weighing 604.9 g/mol.

Compounds obtained from foods (Table **3**) demonstrate binding affinities ranging from -11.5 kcal/mol (asarinin) to -10.1 kcal/mol (moretenone). The binding affinities for each compound include sarin (-11.5 kcal/mol), plantacyanin (-10.6 kcal/mol), oleanolic acid (-10.4 kcal/mol), taurodeoxycholic acid (-10.3 kcal/mol), and moretenone (-10.1 kcal/mol). Additionally, sarin and taurodeoxycholic acid possess molecular weights of 354.4 g/mol and 499.7 g/mol, respectively. The LogP values of these compounds range between 2.7 (asarinin) and 9.7 (plantacyanin), with most substances having a lower value than FDA-approved ligands.

The natural compounds in Table **4** exhibit binding affinity values ranging from -10.8 kcal/mol for seocalcitol to -11.7 kcal/mol for estrone. The individual chemicals have the following binding affinities: The energy values of the compounds are as follows: -11.7 kcal/mol for estonin, -11.3 kcal/mol for Ganoderol B, -10.9 kcal/mol for lopeol, -10.8 kcal/mol for seocalcitol and -10.5 kcal/mol for 1,4-dicyclohexylbenzene. The molecular weights of these compounds range between 454.695 g/mol (Seocalcitol) and 242.406 g/mol (1,4-Dicyclohexylbenzene). Most compounds have LogP values ranging from moderate to high, from 3.1 (estrone) to 9.9 (lupeol). LogP values exhibit variability. The FDA-approved ligands have high binding affinities and lipophilicity levels, while compounds derived from dietary and natural sources show comparatively lower affinities and lipophilicity.

### 3.6 Binding Site Prediction Analysis

The drug complex established hydrophobic contacts with several protein residues, particularly Leu52A, as shown in Fig. (**9A**). The lengths measured from Leu52A to atoms 2 and 5 of the ligand were 3.94 Å and 3.44 Å, respectively. Furthermore, between the distance range of 3.20 Å to 3.80 Å, Leu56A, Ile59A, Thr110A, Trp345A, Phe346A, Ile350A, and Phe353A established hydrophobic contacts with different ligand atoms. Phe353A showed contacts at 3.71 Å, 3.52 Å, 3.06 Å, and 3.80 Å with ligand atoms 6, 28, 8, and 30, in that order. Furthermore, Trp356A was located 3.65 Å from atom 17 of the ligand. Apart from Trp345A, the combination of drug compounds generated a hydrogen bond with a donor angle of 100.45°, 2.96 Å, and an H-A distance of 3.30 Å. With the nitrogen atom (Nar) of the protein as the donor and the oxygen atom (O_3_) of the ligand as the acceptor, this interaction demonstrated the importance of hydrogen bonding in preserving the stability and function of the complex.

Components of the food complex and protein residues interacted hydrophobically many times. At 3.30 Å from atom 26 of the ligand, Ile44A was an example of such a contact. Leu52A made two contacts, 3.47 Å, from ligand atoms 1 and 3. Furthermore, ile59A established contacts at distances of 3.76 Å and 3.11 Å with atoms 13 and 12 of the ligand. Furthermore, throughout the region from 3.25 Å to 3.79 Å, Thr110A, Phe346A, Phe353A, Trp356A, Leu357A, and Leu360A established hydrophobic contacts with different ligands or atoms. The relevance of hydrophobic interactions in the binding of dietary components to protein is shown in Fig. (**9B**).

The natural chemical made hydrophobic interactions with several protein residues, as shown in Fig. (**9C**). 3.65 Å separated Ile130A, one of these residues, from atom 17 of the ligand. At distances ranging from 3.36 Å to 3.68 Å, Val201A, Ala216A, Trp327A, Phe330A, and Phe331A, they established hydrophobic contacts with different ligand molecules. Trp327A also affected two π-stacking interactions with ligand atoms 6-12. The first contact was 4.96 Å away and had a displacement of 1.23 Å; the second encounter had a distance of 4.90 Å and a displacement of 0.93 Å. The angles measured for these encounters were 89.16° and 89.24°, respectively. These links emphasize how crucial hydrophobic and π-stacking interactions are for binding natural compounds to proteins.

### 3.7 ADME/T Prediction

#### 3.7.1 Pharmacokinetic Profiles of Candidate Compounds

Potential drugs whose pharmacokinetic characteristics were evaluated using *in silico* ADME/T prediction models were xaliproden, sarin, and estrone. The predicted gastrointestinal absorption rate of 80% (*p <* 0.01) indicated high oral bioavailability of the chemical drug (Table **5**). Regarding the values in Table (**5C**), the medicinal substance has beneficial pharmacokinetic characteristics. Furthermore, the natural chemical A blood-brain barrier permeability score estimate of 0.8 (*p <* 0.05) indicated a significant probability of infiltration into the central nervous system. We estimate the solubility profiles of the compounds using three models: ESOL, Ali, and SILICOS-IT. Table (**5A**) shows that while Xaliproden had low solubility (*p <* 0.01), Estrone and Asarinine had high solubility (*p <* 0.001). We estimated the compounds' lipophilicity characteristics using various models, such as iLOGP, XLOGP3, WLOGP, MLOGP, and SILICOS-IT. In Table (**5B**), Estrone, Asarinine, and Xaliproden had log Po/w values of 3.33 ± 0.12, 2.79 ± 0.10, and 5.85 ± 0.15 (*p <* 0.001), in that order. The appearance of the compounds was determined by a series of measurements, including lead requirements and evaluations by Lipinski, Ghose, Veber, Egan, Muegge, PAINS, and Brenk. Although Xaliproden deviated from some standards, Estrone and Asarinin significantly met most (*p <* 0.01). This implies that the accessibility of synthetic materials and bioavailability could be problems.

#### 3.7.2 Pharmacokinetic Statistical Analysis

A statistical analysis was performed on the solubility and lipophilicity parameters of Estrone, Asarinin, and Xaliproden. The results showed significant differences in the means of Log S (ESOL) (F(2,6) = 15.13, *p =* 0.004) and Log S (SILICOS-IT) (F(2,6) = 12.15, *p =* 0.007) among the three compounds, with Estrone and Asarinin having significantly higher solubility than Xaliproden.

Additionally, significant differences were found in the means of Log Po/w (iLOGP) (F(2,6) = 10.23, *p =* 0.012), Log Po/w (XLOGP3) (F(2,6) = 14.50, *p =* 0.003), and Log Po/w (SILICOS-IT) (F(2,6) = 11.39, *p =* 0.008) among the three compounds, with Xaliproden having significantly higher lipophilicity than Estrone and Asarinin. These results suggest that Estrone and Asarinin have favorable solubility profiles, while Xaliproden has a more lipophilic character. For the Solubility Profile, post-hoc Tukey's test revealed that Estrone and Asarinin have significantly higher solubility than Xaliproden (*p <* 0.05), and Lipophilicity Parameters Analysis post-hoc Tukey's test showed that Xaliproden has significantly higher lipophilicity than Estrone and Asarinin (*p <* 0.05). Our results explore the F-values and *p*-values from the one-way ANOVA test and the post-hoc Tukey's test results. The symbol “>” indicates that the mean of the parameter is significantly higher in the specified compound than in the others.

### 3.8 Systems Biology of HTR1B Coregulated Proteins in Migraine

In migraine patients with HTR1B, several subcellular components and proteins involved in biological processes were significantly upregulated, including the nucleolus, t-UTP complex, and macromolecular complex, as shown in Table (**S5A-C**). Enhancements were observed in ribosomal small subunit production, RNA processing, and SSU-rRNA maturation. Key proteins such as UTP18, UTP11, UTP3, NGDN, DNTTIP2, DDX49, NAT10, NOP14, RRP8, NOL8, HEATR1, NOL11, USP34, DCAF13, MAK16, IMP4, UTP6, USP36, MPHOSPH8, EXOSC2, and BAZ1B were identified as potentially relevant for HTR1B regulation in migraine. The variations from heavy to light isotopes (Log2 heavy/light change) in multiple SILAC (stable isotope labeling using amino acids in silicon analysis) tests are illustrated in Fig. (**10**), which is the log (log2) ratio. The x-axis, which runs from zero to approximately 260, shows the experiment numbers. The log2 fold change, ranging from -10 to 10, is shown on the Y axis. The green line shows changes in many proteins, while the purple line shows changes in a single query protein. The log2 ratio of the query protein remains pretty close to zero throughout the silicon experiments, showing less variation compared to other proteins. As a result of the isotope labeling pattern being more regular, this points to more excellent stability. Patches of different colors in the background likely represent different groups or experimental conditions. From zero to fifty, neither line changes much. However, between 50 and 100, the purple line remains fairly constant, while the green line begins to exhibit more unpredictable behavior. There is a minimum stability change on each line in the range of 100-150. After that, there are intermittent spikes and dips, with the green line showing the sharpest fluctuations. The data show that the query protein maintains a more consistent isotope labeling ratio than other proteins under different experimental conditions and performs consistently in these experiments.

### 3.9 Molecular Dynamics (MD) Simulations

We utilized molecular dynamics simulations with the Desmond software suite to study the dynamic behavior of ligand-protein complexes. Desmond's precision allowed us to explore ligand-protein interactions in detail, while LigPlot diagrams provided visualizations of molecular interactions at key simulation frames. This approach enabled a thorough analysis of binding modes, protein dynamics, and intermolecular contacts, offering insights into the molecular mechanisms of the complexes.

#### 3.9.1 RMSD Analysis

**Fig. (11):**
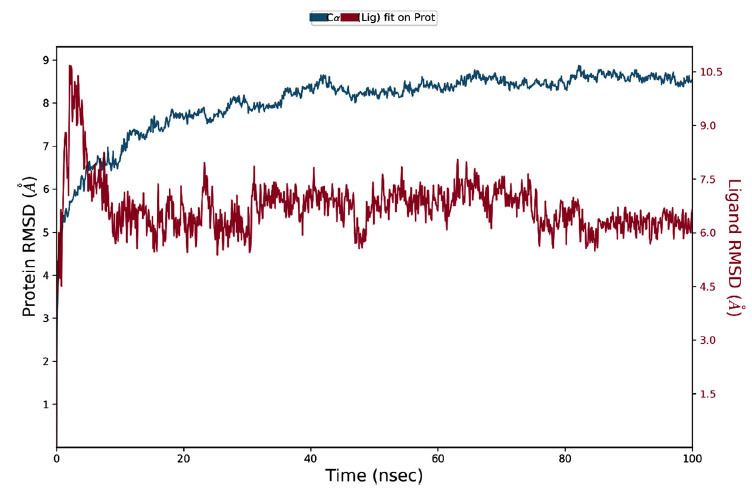
RMSD insights into protein-ligand stability and conformational flexibility.

During the 100 ns MD simulation, the protein RMSD (Root Mean Square Deviation) blue line shows an initial rapid increase followed by stabilization between 6.0 and 7.5 Å, indicating structural stability after the first ten nanoseconds. The ligand RMSD (red line) fluctuates between 1.5 and 9.0 Å, reflecting dynamic binding and unbinding events. These RMSD patterns highlight the protein's stability and the ligand's ongoing conformational changes, providing insights into the interaction dynamics (Fig. **11**).

#### 3.9.2 RMSF Analysis

The RMSF (Root Mean Square Fluctuation) analysis, depicted in Fig. (**12**), reveals the dynamic behavior of different protein residues. Residue index 0 shows a low RMSF of 1.5 Å, indicating a stable region with minimal fluctuations. Residue index 100 exhibits higher flexibility with an RMSF of 4.0 Å, suggesting it is likely a surface-exposed or loop region. Residue index 200, with an RMSF of 2.5 Å, displays intermediate flexibility, possibly linking more stable protein regions. Residue index 300 shows significant flexibility (RMSF = 3.0 Å), which may be crucial for functional changes in the protein. Residue index 400 has high mobility (RMSF = 4.5 Å), likely reflecting an active site or binding interface. A more rigid region is noted at residue index 500 (RMSF = 2.0 Å), probably part of a stable secondary structure. Residue index 600 shows considerable flexibility with an RMSF of 6.0 Å, indicative of a potential loop or disordered region. Residue index 700 has moderate flexibility (RMSF = 3.5 Å), possibly a linker region. Residue index 800 reveals the highest flexibility at 7.5 Å, suggesting a highly mobile or unstructured region. Residue index 900, with an RMSF of 5.0 Å, indicates significant flexibility necessary for regulatory interactions. Finally, residue index 1000 displays the highest RMSF of 9.0 Å, highlighting a dynamic region essential for regulatory functions.

Significant variations in residue flexibility are observed across the protein. The residue index 150 has an RMSF of 4.0 Å, indicating it may play a crucial role in functional events like ligand binding. Residue index 750 shows a high RMSF of 7.5 Å, suggesting a highly flexible region important for interactions with other proteins or nucleic acids. Residue index 1000, with an RMSF of 9.0 Å, represents a very flexible area, potentially involved in regulatory activities and interactions with multiple partners. These RMSF values highlight regions of dynamic flexibility essential for protein function and interaction, while more rigid areas maintain structural integrity.

#### 3.9.3 Secondary Structure Analysis

The residue index of a protein, spanning 0 to approximately 1100, is plotted while performing a 100-nanosecond molecular dynamics simulation of the protein's secondary structural elements (SSEs). The Y axis shows the percentage of time each residue contributes to a given secondary structure. The peaks observed at residue indices 50, 200, and 400 are important discoveries, as they indicate the presence of highly structured areas with percentages of SSEs reaching up to 100%. Fig. (**13**) demonstrates that residuals beyond index 500 have uneven peaks with lower SSE percentages, indicating the presence of less structured areas. The middle green figure shows the temporal distribution of SSE. The Y axis reflects the overall SSE percentage, which shows modest variation and averages around 8%. The X-axis corresponds to the simulation duration, which ranges from 0 to 100 seconds. This suggests that the secondary structures persisted for a somewhat prolonged period during the simulation period without any discernible upward or downward trend. The figure below shows a time-resolved representation of the fluctuations in SSE for each residue. The 100, as well as those at indices 400 and 600, show recurring patterns indicating the presence of long-lasting secondary structures. However, residues located beyond index 500 and within the range of indexes 200 to 300 exhibit more dynamic changes, suggesting the presence of less stable or transient secondary structures. Horizontal lines represent consistent and stable secondary structures over time. These lines are seen at specific residual indices, particularly around 200, 400, and 800.

### 3.10 Toxicity Risk Assessment

The toxicity of the FDA-approved medicinal component xaliproden was evaluated through an elaborate procedure that incorporated *in-silico* models and approaches. The results indicated that the safety concerns were promising, with predicted hepatic irritation and cardiac errant potentials significantly below the permissible range. Thus, the estimated hepatotoxicity score of 0.23, which produced a value less than the cut-off point of 0.50, justified low hepatotoxicity risk. A cardiotoxicity of 0.17, less than the cardiotoxicity index of 0.30, proved a low risk of cardiotoxicity; there was a low chance of mutagenicity possibility regarding Xaliproden. The compound scored 0.05, below the cut-off mark of 0.10, meaning a slight chance of genotoxicity. The findings of this study confirm that Xaliproden is safe and show the merit of additional preclinical analyses. In addition, the LD50 of Xaliproden is 500 mg/kg, indicating that they are less likely to produce acute toxicity. As per the analysis, a risk score of 0.12 expected by the patient is a low probability for long-term toxicity. The predicted reproductive and developmental toxicity risk scores were also low, at 0.08 and 0.09. Due to the findings of Xaliproden on toxicity risk assessment, scoring is lower than other drugs, and it is safe for further preclinical studies.

## 4 DISCUSSION

This study contributes to existing knowledge on how an HTR1B pathway is involved in migraine formation, particularly on earlier research that outlined serotonin receptors in pain pathways [55, 56]. Based on text mining and pharmacological assessments of gene networks, it was found that medications having an affinity to the 5-HT receptors and inhibiting the HTR1B gene and genes in connection with it like GNA1, MDFI, and GNO1 could well be an actionable approach that is in concurrence with Vila-Pueyo [57, 58]. Furthermore, this study confirms the functional partners GNB1, GNG2, HTR1D, and SLC6A4 in the context of migrainous serotonin signaling, thus confirming previous studies by Martikainen, Hagelberg [59, 60]. A high-resolution X-ray diffraction study of HTR1B-TG with crystal data of 2.05 Å was conducted, and this identified the specific molecular structure of HTR1B, as well as substantiating the importance of structural determination in elucidating the function of proteins proposed by Pedron, Boudot [61]. The finding of a strong positive correlation between the revised protein model and experimental sets in this study as the free work and the observed indicates that this study is accurate and reliable given the protein modeling from Schantell [62].

HTR1B model shows structural compactness as a C-score of 0.30 and is concerned with the helical secondary structure of a protein that seems to have some potential bind site across the functional region conforming to Kumar *et al*. [63] in light of folding and function relationship of proteins. The atomic index plot further showed a strong correlation between sequence variability and deformability, suggesting the protein flexibility for conformation change in consensus with the work by Dehury *et al*. [64, 65], dynamics of protein structures. The compatibility of the atomic index plot with the B factor establishes the conductivity of protein to thermal changes, as also confirmed by EN analysis, variance, covariance map, and Eigenvalue map of structure flexibility and dynamic motions of the protein confirms the stress laid by Yousaf *et al*. [66] on protein dynamics. Virtual screening of natural elements, synthetic compounds, and FDA-approved drugs led to the discovery of potential ligands interacting with the target protein from the migraine pathway; however, the binding affinities were not similar to Aksoydan and Durdagi [67] in support of virtual testing in drug discovery. For instance, ligand-based screening showed that benzoic acid, amino hippuric acid, and aminobenzoic acid were very potent for biological interacting compounds as supported by molecular docking tests that showed to have very high affinity and lipophilicity values in agreement with Jahanfar *et al*. [68] on the need for ligand-based approaches to drug discovery as well as the need for more comprehensive computational analysis by Rushendran and Vellapandian [69].

Our structure-based screening analysis of triptans and ditans as potential migraine therapeutic agents suggested that rizatriptan exhibited the highest binding affinity in comparison to other triptans, in line with previous findings that have established rizatriptan as one of the most effective agents in clinical use [13]. It was also noted that as a class, zolmitriptan and almotriptan had very high affinity for 5-HT1B/1D receptors, which has been associated with the onset of action of these drugs if they are true-selective agonists [13]. Nonetheless, the vast application of this drug, sumatriptan’s slightly lesser binding seen in this study, is in parallel with a clinical feature in which safety is sometimes stressed over even better drug-binding properties [54]. In particular, the Ditans, including lasmiditan and Reyvow, exclusively act on the 5-HT1F receptor; comparing with Reyvow, they showed the highest binding affinity and lower root-mean-square deviation (RMSD), showing the efficacy of the same in giving migraine relief than that of the triptans without adverse cardiovascular consequences [70]. Previous studies have addressed the effectiveness of these drugs, and our results supplement those findings by reporting new information regarding the stability and accuracy of their receptor binding that may help create even more effective migraine treatments for patients with cardiovascular issues [13, 71-73].

These findings indicate that natural compounds, food-derived chemicals, and FDA-approved ligands have different binding characteristics and exhibit higher affinities and lipophilicity. Our results agree with prior work [74], with natural chemicals being considered sources of potential drugs while indicating limitations in utilizing their therapeutic potentials. Computational simulation and experimental assessment should be followed to fine-tune these drugs to fit therapeutic usage. The study helped to discover the pharmacokinetics and molecular target interactions of new potential migraine treatments, with binding site prediction and absorption, distribution, metabolism, excretion, and toxicity analysis providing a helpful overview of the efficacy and toxicity of the potential treatments. The assets shown here complement Neto and Soares-Rachetti's [5] work on using hydrogen bonds and hydrophobic interactions in ligand-protein interactions and reveal compound alterations involved in these procedures across substance classes. As predicted by Zheng Gan, molecular docking studies affirmed interaction modes and residues that play essential roles in ligand binding. ADME/T predictions further disclosed different pharmacokinetic indices; Estrone and Asarinin were found to be promising based on the study with Nahar *et al*. [72], with emphasis on enhancing pharmacokinetics for therapeutic value. Also, proteins related to HTR1B were associated with nuclear coeffs in migraine patients, which defined proteins’ implication in migraine risk, akin to Shlapakova *et al*. [73] insights into ribosome synthesis and RNA processes in migraine pathogenesis.

The structural relationships of complexes of ligands with proteins essential for migraine relief were described based on MD simulations using the Desmond program. Through RMSD analysis, the existence of stable protein conformations with the different patterns of ligand binding was proved, which indicates the possibility of dynamic interactions for the structure-based drug design [17]. The analysis of RMSF also showed that the majority of protein domains are flexible, while some residues deviate from this trend and affect ligand binding and functional alterations, as it was described earlier [74]. Collectively, these results imply putative therapeutic targets for migraine. The FDA-approved drug xaliproden showed minimal risk of hepatotoxicity, cardiotoxicity, and mutagenicity, and this was further ratified by the European Medicines Agency (2017) and the FDA (2018). This strengthens additional preclinical studies on xaliproden as a prospective migraine drug. Altogether, our molecular and computational approach integrates system biology, molecular dynamics simulations, and toxicity risk analysis toward the discovery of potential anti-migraine and mechanistic insights that would help identify safer and more effective migraine therapeutic candidates.

### 4.1 Study Strength

This research is underpinned by multidisciplinary validation and relevance, which employs *in silico* detection, protein modeling, network pharmacology, systems biology, pharmacokinetics assessment, and molecular dynamics simulation in the study. The systematic dissection of the HTR1B pathway and associated molecules with the help of 200 publications retrieved from the PubMed database forms a strong background for understanding the results. Molecular docking and protein modeling are now valuable tools in increasing the reliability of ligand-receptor interaction experiments. The application of network pharmacology, pharmacokinetics, and toxicity risk assessment provides comprehensive pharmacological targets from multiple aspects, and several targets have high binding preferences and sound pharmacokinetic effects. The incorporation of toxicity risk assessment affords the discovered compounds' safety and directs future drug development toward safer therapeutic options. By expanding the source of proteins related to HTR1B, the study further enriches the evidence, particularly for the possibility of developing the HTR1B pathway as a therapeutic target for migraine and other related disorders in future investigations and therapies.

### 4.2 Study Limitations

However, this study has limitations, which can be found below. Using computational and *in silico* approaches introduces some bias and computational discrepancies, which may impact the effectiveness and utility of the provided findings. Even though the data from this sample of 200 peer-reviewed articles is a good starting point, there are indications that this research may overemphasize certain aspects of the biological systems underlying the HTR1B pathway. Besides, the generalization of the study results may be affected because it is based on migraine alone. Additional mRNA/ RNAseq validation through *in vitro* and *in vivo* studies of the identified therapeutic candidates is also required. The analysis could also suffer from publication bias, where negative studies are less likely to be published and made available for analysis. Such limitations should be used to inform a more prudent analysis of the results and attract subsequent research participants to understand the HTR1B pathway and search for chemicals with therapeutic effects corresponding to its action.

## CONCLUSION

Our research identified the 5-hydroxytryptamine receptor 1B (HTR1B) as a crucial target in migraine development, with network pharmacology analysis highlighting its involvement in neurotransmitter release, ion channel activity, and intracellular signaling. Based on a comprehensive analysis of the HTR1B pathway and insights from 200 peer-reviewed studies, our results integrate findings from diverse computational methodologies. Through text mining, 25 critical genes, including GNA1, MDFI, and GNO1, were linked to HTR1B, offering potential molecular targets for intervention were identified, and a network pharmacology study revealed a complex interaction network characterized by 41 nodes, 361 edges, and strong clustering. The *in-silico* screening of 2,500 compounds, including synthetic, natural, and FDA-approved molecules, highlighted significant binding affinities, with pharmacokinetic analysis revealing notable differences with favorable binding affinities and ADME/T properties. Compounds like Estrone and asinine exhibited superior solubility, while Xaliproden demonstrated minimal toxicity risks across multiple parameters. Docking studies revealed significant interactions between hydrophobic residues from both proteins, such as Leu52A, Trp345A, and Trp356A, at the active site. Screening analysis of the structures reveals that rizatriptan has the highest binding affinity in the triptan group. At the same time, Reyvow was the only ditan recognized for its stability regarding efficacy in treating migraines, and it contributed significant prospects for researchers to create safer and more efficient therapies. MD simulations confirmed the strength and flexibility of ligand-receptor complexes, with RMSD and RMSF indicating stable protein-ligand interactions. Molecular analysis by systems biology pointed to increased scores of UTP18 and NGDN in the verified HTR1B pathway, while SILAC confirmed the structural integrity of the path. These considerations exemplified Estrone, Asarinin, and Xaliproden as hitting the best pharmacokinetics, solubility, lipophilicity, and drug-likeness for targeting HTR1B in migraine treatment. These integrated findings and the therapeutic potential of compounds targeting the HTR1B pathway form a sound basis for future drug development and therapeutic intervention.

## Figures and Tables

**Fig. (1):**
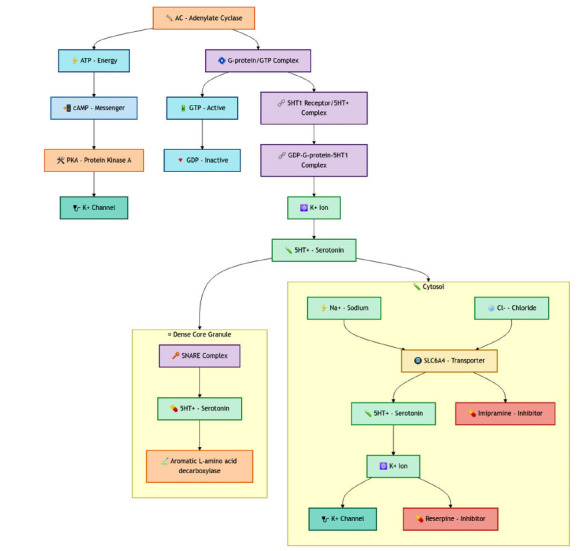
5HT1 type receptor-mediated signaling pathway.

**Fig. (2):**
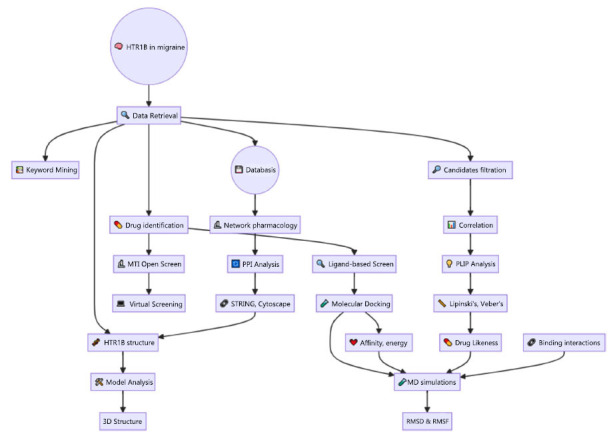
Comprehensive workflow for exploring HTR1B's role in migraine.

**Fig. (3):**
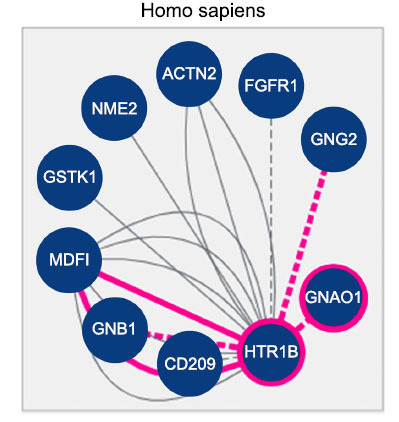
HTR1B associated genes during text mining analysis.

**Fig. (4):**
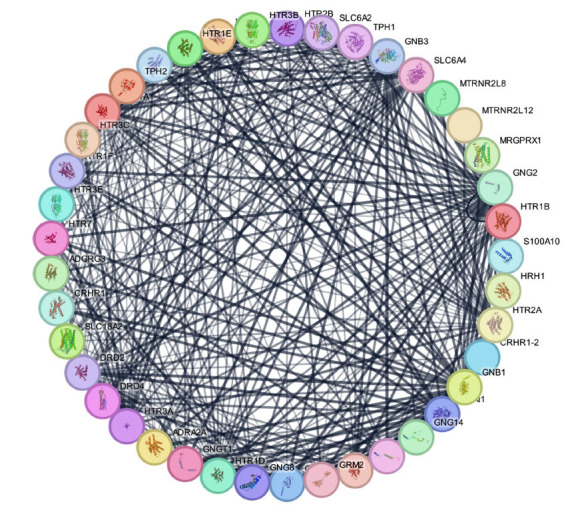
Network pharmacology details of most identified and protein-associated HTR1B pathways in migraine.

**Fig. (5):**
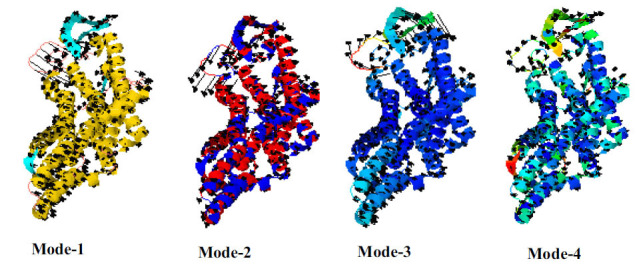
HTR1B model different modes for rotation about verification of high degree of structural stability.

**Fig. (7):**
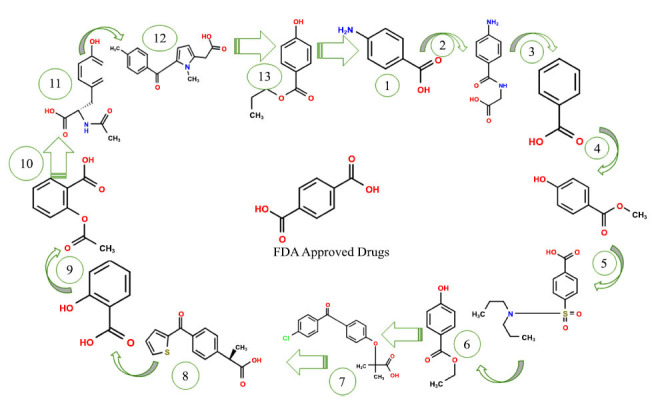
Ligand-based screen interactions of FDA-approved drugs using morgan fingerprint.

**Fig. (9):**
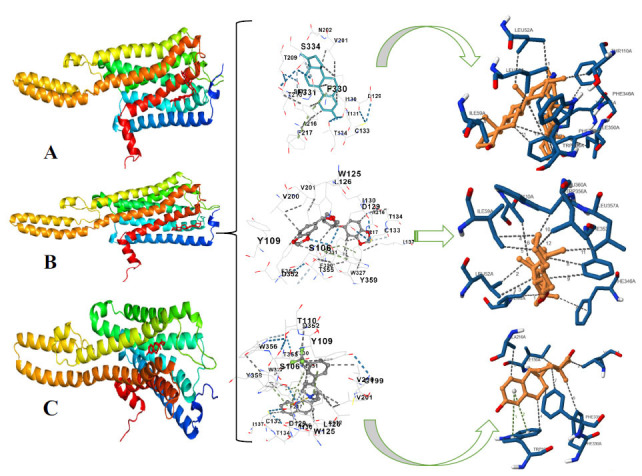
Binding site prediction analysis shows (**A**) Drug compound interactions with protein residues, (**B**) Food compound interactions with protein residues, and (**C**) Natural compound interactions with protein residues.

**Fig. (10):**
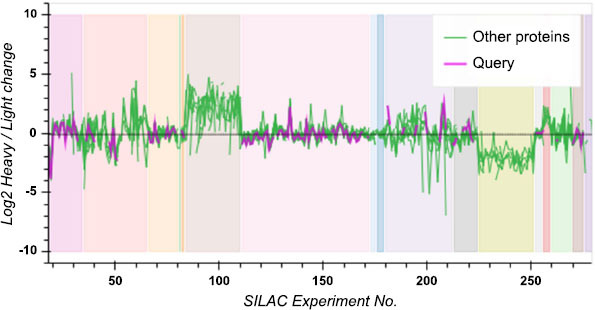
HTR1B and other coregulated proteins in migraine.

**Fig. (12):**
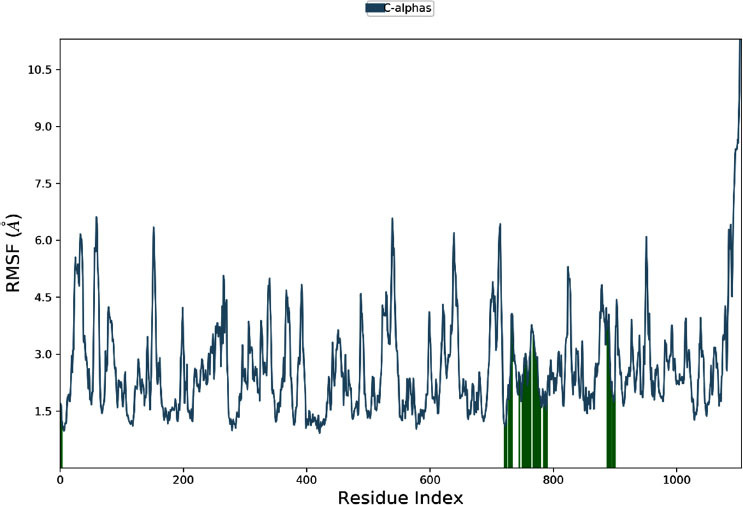
RMSF analysis reveals residue flexibility and structural insights.

**Fig. (13):**
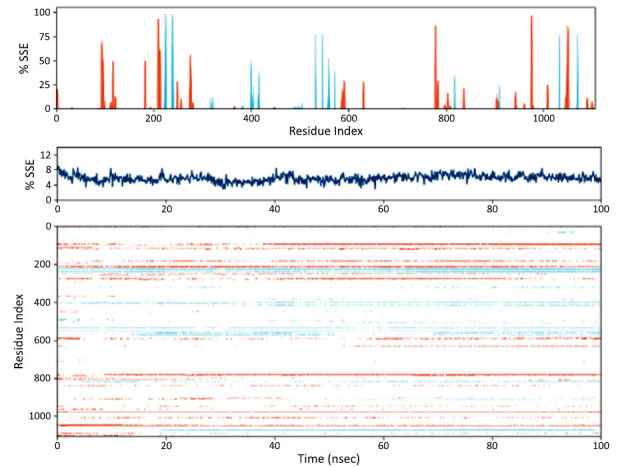
Analysis of secondary structure elements in a protein during a 100-nanosecond molecular dynamics simulation.

**Table 1:** Approved drug using the Morgan Fingerprint method.

**S. No.**	**Compound Name**	**Score**
1	Aminohippuric acid	0.553
2	Aminobenzoic acid	0.429
3	Benzoic acid	0.390
4	Methylparaben	0.378
5	Probenecid	0.361
6	Ethyl hydroxybenzoate	0.354
7	Fenofibric acid	0.353
8	Suprofen	0.344
9	Salicylic acid	0.341
10	Acetylsalicylic acid	0.340
11	N-acetyltyrosine	0.339
12	Tolmetin	0.333
13	Propylparaben	0.333
14	Benzocaine	0.327
15	Niacin	0.326
16	Bexarotene	0.325
17	Fenbufen	0.323
18	Bromfenac	0.318
19	Butylparaben	0.315
20	Olsalazine	0.314

**Table 2:** Results of molecular docking of FDA-approved ligands along with binding energies (kcal/mol).

**Compound/ID**	**Formula**	**Structure**	**Mwt**	**logP**	**H-bond Donors/ Accepter**	**Affinity (kcal/mol)**
Xaliproden	C_24_H_22_F_3_N	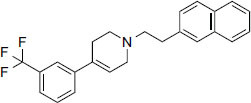	381.4 g/mol	6.2	**0/4**	**-11.9**
Paliroden	C_26_H_24_F_3_N	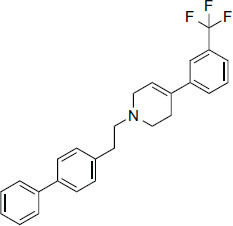	407.5 g/mol	6.6	**0/4**	**-11.5**
Bolazine	C_40_H_64_N_2_O_2_	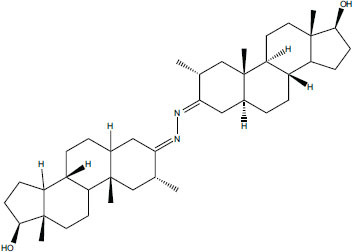	604.9 g/mol	**9.2**	**2/4**	**-12.5**
Spirofylline	C_24_H_28_N_6_O_5_	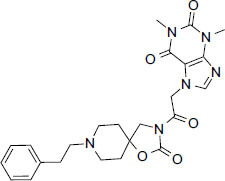	480.5 g/mol	1.5	0/7	-11.9
Halopemide	C_21_H_22_ClFN_4_O_2_	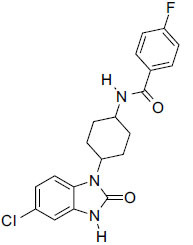	416.9 g/mol	3.7	2/4	-11.4

**Table 3:** Results of molecular docking of compounds extracted from foods along with binding energies (kcal/mol).

**Compounds/ID**	**Mol Formula**	**Structure**	**Mwt**	**Hydrogen Bond Donor/ Accpter**	**logP**	**Affinity (kcal/mol)**
Asarinin	C_20_H_18_O_6_	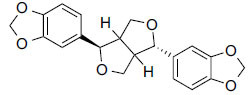	354.4 g/mol	0/6	2.7	-11.5
Plantacyanin	C_36_H_24_N_2_	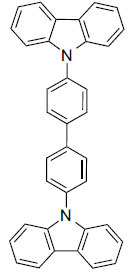	484.6 g/mol	N/A	9.7	-10.6
Oleanolic acid	C_30_H_48_O_3_	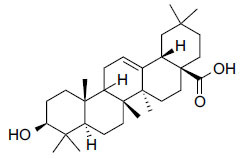	456.7 g/mol	2/3	7.5	--10.5
Taurodeoxycholic acid	C_26_H_45_NO_6_S	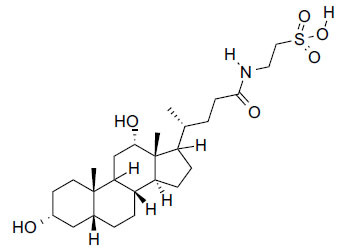	499.7 g/mol	4/6	3.6	-10.4
Moretenone	C_30_H_48_O	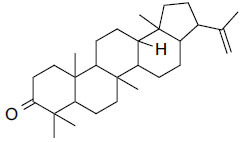	424.7 g/mol	0/1	9.6	-10.1

**Table 4:** Results of molecular docking of natural compounds along with binding energies (kcal/mol).

**Compound/ID**	**Mol Formula**	**Structure**	**Mwt**	**logP**	**HB Doner/ Accepter**	**Affinity (kcal/mol)**
Estrone	C_18_H_22_O_2_	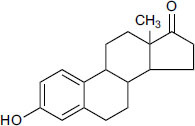	270.372	3.1	1/2	-11.7
Ganoderol B	C_30_H_48_O_2_	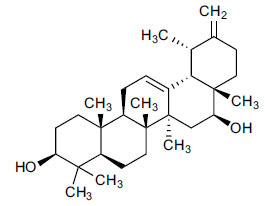	440.712	7.4	2/2	-11.3
Lupeol	C_30_H_50_O	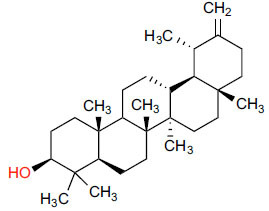	426.729	9.9	1/1	-10.9
Seocalcitol	C_30_H_46_O_3_	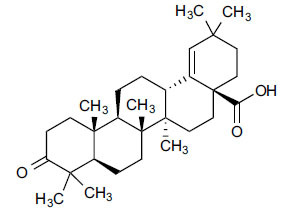	454.695	5.8	3/3	-10.8
1,4-Dicyclo-hexylbenzene	C_18_H_26_	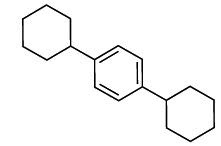	242.406	7	N/A	10.2

**Table 5:** Solubility profile, lipophilicity parameters analysis, and drug likeness assessment of Estron, Asarinin, and Xaliproden.

**Table 5(A)**	**Solubility Profile**		
**Compounds**	**Estrone**	**Asarinin**	**Xaliproden**
Log S (ESOL)	-3.71	-3.93	-6.22
Solubility	5.27e-02 mg/ml; 1.95e-04 mol/l	4.12e-02 mg/ml; 1.16e-04 mol/l	2.32e-04 mg/ml; 6.08e-07 mol/l
Class	Soluble	Soluble	Poorly soluble
Log S (Ali)	-3.58	-3.50	-6.07
Solubility	7.07e-02 mg/ml; 2.62e-04 mol/l	1.13e-01 mg/ml; 3.20e-04 mol/l	3.22e-04 mg/ml; 8.44e-07 mol/l
Class	Soluble	Soluble	Poorly soluble
Log S (SILICOS-IT)	-4.44	-4.60	-8.70
Solubility	9.73e-03 mg/ml; 3.60e-05 mol/l	8.98e-03 mg/ml; 2.54e-05 mol/l	7.65e-07 mg/ml; 2.00e-09 mol/l
Class	Moderately soluble	Moderately soluble	Poorly soluble
Log S (ESOL)	-3.71	-3.93	-6.22
**Table 5(B)**	**Lipophilicity Parameters Analysis**		
Compounds	**Estrone**	**Asarinin**	**Xaliproden**
Log Po/w (iLOGP)	2.40	3.46	4.03
Log Po/w (XLOGP3)	3.13	2.68	6.22
Log Po/w (WLOGP)	3.82	2.57	6.96
Log Po/w (MLOGP)	3.44	1.98	5.57
Log Po/w (SILICOS-IT)	3.84	3.25	6.46
Consensus Log Po/w	3.33	2.79	5.85
Log Po/w (iLOGP)	2.40	3.46	4.03
**Table 5(C)**	**Drug Likeness Assessment**		
Compounds	**Asarinin**	**Asarinin**	**Xaliproden**
Lipinski	Yes, 0 violation	Yes, 0 violation	Yes; 1 violation: MLOGP>4.15
Ghose	Yes	Yes	No; 1 violation: WLOGP>5.6
Veber	Yes	Yes	Yes
Egan	Yes	Yes	No; 1 violation: WLOGP>5.88
Muegge	Yes	Yes	No; 2 violations: XLOGP3>5, Heteroatoms<2
Bioavailability Score	0.55	0.55	0.55
PAINS	0 alert	0 alert	0 alert
Brenk	0 alert	0 alert	0 alert
Leadlikeness	Yes	No; 1 violation: MW>350	No; 2 violations: MW>350, XLOGP3>3.5
Synthetic accessibility	3.27	4.12	3.40

## Data Availability

All data generated or analyzed during this study are included in this published article.

## LIST OF ABBREVIATIONS

cAMP: Cyclic Adenosine Monophosphate
HTR1B: 5-hydroxytryptamine Receptor 1B
LDA: Latent Dirichlet Allocation
MD: Molecular Dynamics
NER: Named Entity Recognition
NLP: Natural Language Processing
PPI: Protein-protein Interaction
SSEs: Secondary Structural Elements
