# Supplementary material for: Informatics Approach Towards Targeting HTR1B Pathways in Neuropharmacology for Migraine Treatment
Source: Curr Neuropharmacol. 2025 Feb 6;23(14):1939–61. doi: 10.2174/011570159X341703250130064735 (PMC12676031; doi:10.2174/011570159X341703250130064735)
Supplement: Supplementary file 1 [file CN-23-14-1939_SD1.pdf]

Supplementary Material

Informatics Approach Towards Targeting HTR1B Pathways in Neuropharmacology for Migraine Treatment

Saleem Ahmad[1], Li Wang[2,3], Imran Zafar[4,*], Zain Abbas[5], Ahsanullah Unar[6], Mohamed Mohany[7], Salim S. Al-Rejaie[7], Najeeb Ullah Khan[8], Ijaz Ali[9] and Muhammad Shafiq[10,*]

[1]Cardiovascular Center of Excellence, Louisiana State University Health Sciences Center, New Orleans, LA, United States; [2]Shenzhen Hospital Beijing University of Chinese Medicine, Guangdong, China; [3]Shenzhen University General Hospital, Shenzhen University, Guangdong, China; [4]Department of Biotechnology, The University of Faisalabad (TUF), Faisalabad, Punjab, Pakistan; [5]Department of Life Sciences, University of Management and Technology, Lahore, Punjab Pakistan; [6]Department of Precision Medicine, University of Campania 'L. Vanvitelli', Naples, Italy; [7]Department of Pharmacology and Toxicology, College of Pharmacy, King Saud University, P.O. Box 55760, Riyadh 11451, Saudi Arabia; [8]Institute of Biotechnology & Genetic Engineering (Health Division), The University of Agriculture, Peshawar, Pakistan; [9]Centre for Applied Mathematics and Bioinformatics (CAMB), Gulf University for Science and Technology, Hawally, Kuwait; [10]Research Institute of Clinical Pharmacy, Department of Pharmacology, Shantou University Medical College, Shantou, 515041, China

1875-6190/25 $65.00+.00 **© 2025 Bentham Science Publishers**

Table S1: Summary of Selected Articles and Data Extraction for HTR1B and Migraine Study.

Compound	Binding Affinity(Kcal/mol)
Plantacyanin_ZINC000022007964	-10.6
5alpha__Campestan_3_one_ZINC000015202068	-9.1
cholesteryl_?_D_glucoside_ZINC000606576795	-7.3
Taurodeoxycholic_acid_ZINC000118916599	-10.4
beta_Amyrin_acetate_ZINC000257358445	-10.5
Fucosterol_ZINC000118915349	-9.3
Fucosterol_ZINC000064219058	-9.8
Sapogenins_ZINC000257348754	-11.5
beta_Amyrenone_ZINC000031165761	-10.6
Daidzein_7_glucuronide_ZINC000503959504	-9
Asarinin_ZINC000002557132	-11.1
17_beta_Estradiol_3_sulfate_17__beta_D_glucuronide__ZINC000616583934	-9.4
Oleanolic_acid_ZINC000017021279	-10.5
20alpha_Hydroxycholesterol_ZINC000004096811	-7.1
Mangostanol_ZINC000013382498	-9.5
Ketoconazole_ZINC000003872994	-6.2
Isoangustone_A_ZINC000014727602	-8.7
Honyucitrin_ZINC000014780845	-7.6
Moretenone_ZINC000002570124	-10.1
Ketoconazole_ZINC000000643138	-6.7

Table S2: The binding affinities of the top 100 FDA-approved drugs with the target protein.

Compound	Binding Affinity(Kcal/mol)
MolPort-039-052-621_ZINC000044404209	-9.9
MolPort-020-005-756_ZINC000031459237	-10.4
MolPort-042-675-382_ZINC000013485410	-8.8
MolPort-001-740-357_ZINC000013374324	-8.7
MolPort-028-754-125_ZINC000008829452	-10.8
MolPort-027-835-585_ZINC000004027386	-10.9
MolPort-039-338-463_ZINC000238774071	-11.3
MolPort-003-911-498_ZINC000253497609	-7
MolPort-001-832-299_ZINC000005356864	-9.7
MolPort-001-740-946_ZINC000004654620	-7.9
MolPort-002-911-113_ZINC000253497992	-6.8
MolPort-001-832-299_ZINC000003869898	-11.7
MolPort-002-507-280_ZINC000003941105	-10.1
MolPort-000-733-658_ZINC000079183226	-7.8
MolPort-006-668-597_ZINC000031169794	-8
MolPort-044-183-452_ZINC000101104515	-9.7
MolPort-005-909-605_ZINC000253388536	-9.6
MolPort-002-893-379_ZINC000001693423	-10.2
MolPort-039-052-311_ZINC000245205019	-9.9
MolPort-002-510-946_ZINC000038140884	-9.6
MolPort-020-005-773_ZINC000238809538	
MolPort-002-527-314_ZINC000004082224	
MolPort-019-992-917_ZINC000006067010	

Table S3: Vina-based virtual screening for food-derived small molecules.

Compound	Binding Affinities(Kcal/mol)
Telcagepant_ZINC000028827350	-10
Xaliproden_ZINC000000577115	-11.9
Paliroden_ZINC000035826853	-11.5
Elinogrel_ZINC000043153259	-8.7
Bolazine_ZINC000008214506	-12.5
Spirofylline_ZINC000084758966	-11.9
Rimacalib_ZINC000004423231	-10.8
Imiglitazar_ZINC000049756486	-7.7
Flibanserin_ZINC000052716421	-8.9
Capmatinib_ZINC000043195321	-8.9
Cinuperone_ZINC000031425112	-8.5
Efatutazone_ZINC000033975065	-7.9
Ag-13958_ZINC000095909141	-7.7
Talniflumate_ZINC000001844627	-8.9
Seganserin_ZINC000000538333	-10.9
Belaperidone_ZINC000003822014	-11
Halopemide_ZINC000000537818	-11.4
Pimozide_ZINC000004175630	-9.5
Rebastinib_ZINC000063933734	-8.1
Ac-430_ZINC000095079936	-8

Table S4: Binding affinity range of the top 100 ligands from the natural compound library.

Compound	Model ID	Energy	nRot
Telcagepant_ZINC000028827350	1	-13.8	5
Xaliproden_ZINC000000577115	1	-13.4	5
Paliroden_ZINC000035826853	1	-13.4	6
Elinogrel_ZINC000043153259	1	-13.0	5
Bolazine_ZINC000008214506	1	-12.5	3
Spirofylline_ZINC000084758966	1	-12.5	5
Rimacalib_ZINC000004423231	1	-12.4	4
Imiglitazar_ZINC000049756486	1	-12.3	11
Flibanserin_ZINC000052716421	1	-12.3	5
Capmatinib_ZINC000043195321	1	-12.1	4
Cinuperone_ZINC000031425112	1	-12.1	6
Efatutazone_ZINC000033975065	1	-12.0	8
Ag-13958_ZINC000095909141	1	-12.0	6
Talniflumate_ZINC000001844627	1	-12.0	6
Seganserin_ZINC000000538333	1	-12.0	5
Belaperidone_ZINC000003822014	1	-12.0	4
Halopemide_ZINC000000537818	1	-11.9	5
Pimozide_ZINC000004175630	1	-11.9	8
Rebastinib_ZINC000063933734	1	-11.9	7
Ac-430_ZINC000095079936	1	-11.9	5
Indopine_ZINC000001482088	1	-11.9	6
Xl-019_ZINC000095930152	1	-11.9	6
Lusaperidone_ZINC000001903948	1	-11.9	3
Ocaperidone_ZINC000000538119	1	-11.8	4
Lidoflazine_ZINC000022034381	1	-11.8	9
Floxacrine_ZINC000001846338	1	-11.8	3
Azd-4769_ZINC000013888387	1	-11.8	11
Ketanserin_ZINC000000537877	1	-11.8	5
Sarizotan_ZINC000000021067	1	-11.7	5
Mk3207_ZINC000103760984	1	-11.7	4
Ziprasidone_ZINC000000538550	1	-11.7	4
Erismodegib_ZINC000068202099	1	-11.7	6
Pimobendan_ZINC000004170129	1	-11.6	3
Posaconazole_ZINC000003938482	1	-11.6	13
Napitane_ZINC000000005009	1	-11.6	3
Flotrenizine_ZINC000031597122	1	-11.6	10
Pimobendan_ZINC000005071680	1	-11.6	3
Capeserod_ZINC000000603773	1	-11.6	6
Lorpiprazole_ZINC000033827894	1	-11.6	5

Compound	Model ID	Energy	nRot
Balaglitazone_ZINC000001481805	1	-11.6	5
Cloperidone_ZINC000004215426	1	-11.5	5
Xl-019_ZINC000095560529	1	-11.5	6
Fluazuron_ZINC000002570819	1	-11.5	5
Dexetimide_ZINC000006927490	1	-11.5	4
Enpiroline_ZINC000004628938	1	-11.5	6
Nebivolol_ZINC000004213946	1	-11.5	8
Lemborexant_ZINC000118073503	1	-11.5	6
Enpiroline_ZINC000001568036	1	-11.5	6
Siponimod_ZINC000006717453	1	-11.5	10
Tecarfarin_ZINC000013985547	1	-11.5	7
Doconazole_ZINC000001567316	1	-11.4	7
Piriqualone_ZINC000004217322	1	-11.4	3
Gsk163090_ZINC000034451922	1	-11.4	5
Netoglitazone_ZINC000003633833	1	-11.4	5
Dihydroergocristine_ZINC000095862766	1	-11.4	6
Bifonazole_ZINC000003812958	1	-11.3	4
Bilastine_ZINC000003822702	1	-11.3	10
Ag-24322_ZINC000200158122	1	-11.3	5
Adapalene_ZINC000003784182	1	-11.3	4
Englitazone_ZINC000005933614	1	-11.3	4
Setoperone_ZINC000000538339	1	-11.3	5
Taprenepag_ZINC000072266311	1	-11.3	10
Tobuterol_ZINC000031495045	1	-11.3	11
Etriciguat_ZINC000000603759	1	-11.3	5
Englitazone_ZINC000005933617	1	-11.3	4
Etibendazole_ZINC000005386462	1	-11.3	4
Ergotamine_ZINC000052955754	1	-11.3	5
Lifarizine_ZINC000019368434	1	-11.2	6
Tesevatinib_ZINC000038912363	1	-11.2	6
Acodazole_ZINC000018009339	1	-11.2	3
Omarigliptin_ZINC000084758480	1	-11.2	4
Enpiroline_ZINC000001568034	1	-11.2	6
Ontazolast_ZINC000000005761	1	-11.2	5
Edaglitazone_ZINC000001483899	1	-11.2	7
Quinaldine-blue_ZINC000001687632	1	-11.2	4
Phthalylsulfamethizole_ZINC000033852268	1	-11.2	6
Sb-705498_ZINC000034297799	1	-11.2	4
Enpiroline_ZINC000001568035	1	-11.2	6
Monatepil_ZINC000000607979	1	-11.2	6
Balaglitazone_ZINC000001489816	1	-11.2	5

Compound	Model ID	Energy	nRot
Telatinib_ZINC000000590964	1	-11.2	6
Prenoxdiazine_ZINC000000538227	1	-11.2	7
Mk3207_ZINC000103760981	1	-11.2	4
Flufylline_ZINC000000537738	1	-11.1	5
Arpromidine_ZINC000014951255	1	-11.1	9
Ph-797804_ZINC000013980453	1	-11.1	5
Glipizide_ZINC000000537795	1	-11.1	9
Englitazone_ZINC000005933664	1	-11.1	4
Venetoclax_ZINC000150338755	1	-11.1	13
Flezelastine_ZINC000003785445	1	-11.1	6
Derenofylline_ZINC000252673365	1	-11.1	4
Entrectinib_ZINC000043204146	1	-11.1	7
Dihydroergocristine_ZINC000003947495	1	-11.1	6
Tesevatinib_ZINC000117147301	1	-11.1	6
Losmapimod_ZINC000035793138	1	-11.1	6
Devazepide_ZINC000001847292	1	-11.1	3
Spirilene_ZINC000000538370	1	-11.1	5
Entinostat_ZINC000001488870	1	-11.0	8
Florifenine_ZINC000000537734	1	-11.0	8
Englitazone_ZINC000005933666	1	-11.0	4

Table S5(A): GO Subcellular Components enriched among the currently selected co-regulated proteins.

Subcellular Components	No. of proteins	Adj p-value
nucleolus	21	8.17E-24
small-subunit processome	10	5.79E-20
nuclear lumen	21	1.77E-11
90S pre ribosome	6	2.87E-10
nucleus	22	1.08E-6
nucleolar part	5	8.56E-6
t-UTP complex	2	1.29E-2
macromolecular complex	15	1.44E-2
Pwp2p-containing subcomplex of 90S pre ribosome	2	3.23E-2

Table S5(B): GO Biological Processes enriched among the currently selected co-regulated proteins.

Biological Processes	No. of proteins	Adj p-value)
rRNA processing	15	1.19E-23
ribosome biogenesis	15	1.76E-21
maturation of SSU-rRNA	8	4.09E-14
ribosomal small subunit biogenesis	8	6.39E-13
maturation of SSU-rRNA from tricistronic rRNA transcript (SSU-rRNA, 5.8S rRNA, LSU-rRNA)	7	2.71E-12
RNA metabolic process	18	2.18E-6
gene expression	17	2.12E-4
cellular macromolecule metabolic process	20	7.5E-4
maturation of 5.8S rRNA	3	2.2E-2
metabolic process	21	4.45E-2
primary metabolic process	20	4.76E-2

Table S5(C): Protein Network associations with 99% confidence.

Uniprot Acc	Gene Name	Protein Name	Percentile Score
Q9Y5J1	UTP18	U3 small nucleolar RNA-associated protein 18 homolog	0.998812
Q9Y3A2	UTP11	Probable U3 small nucleolar RNA-associated protein 11	0.998661
Q9NQZ2	UTP3	Something about silencing protein 10	0.9982
Q8NEJ9-2	NGDN	Neuroguidin	0.998056
Q5QJE6	DNTTIP2	Deoxynucleotidyltransferase terminal-interacting protein 2	0.997931
Q9Y6V7	DDX49	Probable ATP-dependent RNA helicase DDX49	0.997731
Q9H0A0	NAT10	RNA cytidine acetyltransferase	0.997314
P78316	NOP14	Nucleolar protein 14	0.996825
O43159	RRP8	Ribosomal RNA-processing protein 8	0.99675
Q76FK4-4	NOL8	Nucleolar protein 8	0.996649
Q9H583	HEATR1	HEAT repeat-containing protein 1	0.996649
Q9H8H0	NOL11	Nucleolar protein 11	0.996579
Q70CQ2	USP34	Ubiquitin carboxyl-terminal hydrolase 34	0.996101
Q9NV06	DCAF13	DDB1- and CUL4-associated factor 13	0.996092
Q9BXY0	MAK16	Protein MAK16 homolog	0.99583
Q96G21	IMP4	U3 small nucleolar ribonucleoprotein protein IMP4	0.995646
Q9NYH9	UTP6	U3 small nucleolar RNA-associated protein 6 homolog	0.995462
Q9P275	USP36	Ubiquitin carboxyl-terminal hydrolase 36	0.99528
Q99549	MPHOSPH8	M-phase phosphoprotein 8	0.995238
Q13868	EXOSC2	Exosome complex component RRP4	0.995212
Q9UIG0	BAZ1B	Tyrosine-protein kinase BAZ1B	0.995153
Q9NPD3	EXOSC4	Exosome complex component RRP41	0.995149
Q8IWA0	WDR75	WD repeat-containing protein 75	0.995
